# The value of the ACEF II score in Chinese patients with elective and non-elective cardiac surgery

**DOI:** 10.1186/s12872-022-02946-6

**Published:** 2022-12-02

**Authors:** Zhiming Mo, Penghua Hu, Zhiyong Xie, Yanhua Wu, Zhilian Li, Lei Fu, Yuanhan Chen, Xinling Liang, Huaban Liang, Wei Dong

**Affiliations:** 1 The Second School of Clinical Medicine, Southern Medical University, Guangzhou, China; 2 Department of Nephrology, Guangdong Provincial People’s Hospital, Guangdong Academy of Medical Sciences, Guangzhou, China; 3 Division of Nephrology, The Affiliated Yixing Hospital of Jiangsu University, Yixing, China

**Keywords:** Cardiac surgery, Emergency surgery, Risk assessment, Death, Acute kidney injury

## Abstract

**Objective:**

To evaluate the value of the ACEF II score in predicting postoperative hospital death and acute kidney injury requiring dialysis (AKI-D) in Chinese patients.

**Methods:**

This retrospective study included adult patients who underwent cardiopulmonary bypass open heart surgery between January 2010 and December 2015 at Guangdong Provincial People’s Hospital. ACEF II was evaluated to predict in-hospital death and AKI-D using the Hosmer–Lemeshow goodness of fit test for calibration and area under the receiver operating characteristic (ROC) curve for discrimination in non-elective and elective cardiac surgery.

**Results:**

A total of 9748 patients were included. Among them, 1080 underwent non-elective surgery, and 8615 underwent elective surgery. Mortality was 1.8% (177/9748). In elective surgery, the area under the ROC (AUC) of the ACEF II score was 0.704 (95% CI: 0.648–0.759), similar to the ACEF score of 0.709 (95% CI: 0.654–0.763). In non-elective surgery, the AUC of the ACEF II score was 0.725 (95% CI: 0.663–0.787), higher than the ACEF score (AUC = 0.625, 95% CI: 0.553–0.697). The incidence of AKI-D was 3.5% (345/9748). The AUC of the ACEF II score was 0.718 (95% CI: 0.687–0.749), higher than the ACEF score (AUC = 0.626, 95% CI: 0.594–0.658).

**Conclusion:**

ACEF and ACEF II have poor discrimination ability in predicting AKI-D in non-elective surgery. The ACEF II and ACEF scores have the same ability to predict in-hospital death in elective cardiac surgery, and the ACEF II score is better in non-elective surgery. The ACEF II score can be used to assess the risk of AKI-D in elective surgery in Chinese adults.

**Supplementary Information:**

The online version contains supplementary material available at 10.1186/s12872-022-02946-6.

## Introduction

Ischemic heart disease is a leading cause of death in many developed and developing countries [1]. Many patients require open-heart surgery, but they are at substantial risk of complications, including pulmonary complications (33%), delirium (26%), and arrhythmias (30%) [2–4], especially the elderly patients. These complications prolong hospitalization, lead to readmission, and increase healthcare costs [5–8]. The overall mortality after open-heart surgery is 1-3% [9, 10] and is even higher in patients who develop postoperative acute kidney injury (AKI) [11, 12]. AKI requiring dialysis (AKI-D) is an independent risk factor for death, and the survival of patients with AKI-D after open-heart surgery remains dismal [12].

Multiple attempts at therapeutic interventions failed to demonstrate benefits in improving renal injury or survival. Successful interventions suggested that intervention should be performed early, within 24 to 48 h after AKI. Still, clinical trials of such early interventions are difficult because it is difficult to anticipate AKI [13, 14]. In addition, the use of biomarkers such as estimated glomerular filtration rate (eGFR) leads to a delay in diagnosis.

Risk stratification is used to predict AKI and identify patients at risk of poor outcomes. There are several models predicting AKI after cardiac surgery [15–17]. The Cleveland Risk Score [17] was established using 10 variables, but its external validation is limited and has not been validated in China. The European system for cardiac operative risk evaluation (EuroSCORE II) [18], The American Society of Thoracic Surgeons (STS) score [19], and the ACEF score [20] are commonly used to predict AKI and identify patients who are at risk of death after open-heart surgery. The EuroSCORE II [18] was established with 17 variables based on the EuroSCORE. The American Association for Thoracic Surgery established the STS score using 42 risk factors [19]. Still, the EuroSCORE II and STS scores require complicated calculations, variables, and tools. The ACEF score [20] can quickly and easily assess the risk of death within 30 days after surgery using only three variables (age, ejection fraction, and serum creatinine) [21] in patients who underwent elective cardiac surgery; these three factors are independent risk factors of death and postoperative AKI after elective surgery [15–17], but not after non-elective surgery [9]. Ranucci et al. [22] established the ACEF II score by adding emergency surgery and anemia to the ACEF score.

The existing models for AKI-D after cardiac surgery have a poor discriminative ability in Chinese patients since they were established based on European and North American populations. The risk factors of the ACEF and ACEF II models for predicting post-surgery mortality are the same factors also predicting AKI.

We hypothesized that ACEF and ACEF II can be used to predict the occurrence and mortality of AKI after cardiac surgery. Chen SW et al. explained that ACEF can be used to predict all stages of AKI. [23] Chang CH et al. demonstrated that ACEF scores exhibited satisfactory predictive ability for all AKI severities [24]. The performance of ACEF II in predicting death and AKI-D in the Chinese population is unclear.

This study aimed to examine the value of the ACEF II score in predicting in-hospital mortality and postoperative AKI-D in Chinese patients who underwent elective and non-elective cardiac surgery. The results could help improve mortality risk prediction in such patients and identify those needing a closer follow-up.

## Methods

### Patients

This retrospective study included adult patients (> 18 years) who underwent cardiopulmonary bypass open heart surgery between January 2010 and December 2015 at the Department of Cardiac Surgery of Guangdong Provincial People’s Hospital. The patients were identified through the Electronic Health Record System (EHRs). The exclusion criteria were 1) congenital heart disease, 2) end-stage renal disease, 3) heart transplantation, 4) renal replacement therapy, 5) unilateral nephrectomy, or 6) rescue surgery [18] (including cardiopulmonary resuscitation, intraoperative or before anesthesia; only the first surgical episode was considered). This study was approved by the Medical Ethics Committee of Guangdong Provincial People’s Hospital (#GDREC2016194H). The requirement for individual informed consent was waived.

### Data collection

The demographic data (age, sex, hypertension, diabetes, CKD, COPD, CCS class, extracardiac arteriopathy, neurological dysfunction, acute endocarditis, myocardial infarction, and previous cardiac surgery), surgery-related data (ejection fraction, cardiopulmonary bypass (CPB) time, types of cardiac surgery, including coronary artery bypass graft, valve surgery, combined coronary artery bypass graft, and valve procedures, and other cardiac surgeries such as ventricular aneurysm repair, pericardiectomy, thoracic aorta surgery, and combined surgery [25]), and laboratory results (serum creatinine, eGFR, hematocrit, etc.) were collected from the EHRs through structured query language [26] as defined in the EuroSCORE II standard [18]. Creatinine datas were extracted available and the maximal creatinine within 7 days postsurgery was used as the basis for AKI evaluation.The ACEF and ACEF II scores were calculated before surgery.

### Definition

AKI was defined as an increase in serum creatinine (Scr) > 0.3 mg/dl (26.5 µmol/L) or an increase in Scr > 50% (reaching 1.5 times the baseline), or a decrease in urine output (< 0.5 ml/kg/h) for more than 6 h according to the KDIGO clinical practice guidelines [27]. Elective surgery was defined as the patient undergoing surgery ≥ 24 h after admission. Non-elective surgery was defined as the patient undergoing surgery within 24 h after admission. The ACEF score was defined as age/ejection fraction + 1 (serum creatinine ≥ 2.0 mg/ dL). The ACEF II score was defined as age/ejection fraction + 1 (serum creatinine > 2.0 mg/dL) + 3 (emergency surgery) + 0.2 × (36%-hematocrit).Dialysis was defined as the appearance of indications that included uremia (eGFR < 10 ml/min, creatinine > 707 µmol/L), hyperkalemia (serum potassium > 5.5 mmol/L), acidosis (decreased plasma [HCO^−^], or increased plasma [H_2_CO_3_] concentration, or pH showed a decreasing trend), volume overload (renal insufficiency accompanied by obvious edema, pulmonary edema, and cardiac insufficiency) [28], or biochemical abnormalities (such as endogenous creatinine clearance rate < 10 mL/min, urea nitrogen > 28.6 mmol/L, or blood creatinine > 707 μmol/L), due to renal insufficiency and were based on clinical judgment by the nephrologist,cardiac surgeon and ICU consultant.and they also participated in dialysis strategy.

### Outcome

The primary outcome is AKI-D 7 days postsurgery and the secondary outcome is death during hospitalization.AKI-D includes patients who need dialysis and actually received dialysis due to renal insufficiency.

### Statistical analysis

The data were analyzed using SPSS 25.0 (IBM, Armonk, NY, USA) and R (version 3.6.1; https://www.r-project.org). The Kolmogorov–Smirnov test was used to assess the continuous variables for normal distribution. The continuous variables with a normal distribution (or non-normal distribution) were described as means ± standard deviation (or medians [range]), and the differences between groups were analyzed using the analysis of variance (or the rank-sum test). The categorical variables were described as n (%) and analyzed using the chi-square test. The multiple imputations by chained equations were used to deal with the missing data of variables, including hematocrit (0.08%), ejection fraction (4.87%), and cardiopulmonary bypass time (0.42%) [29, 30]. The ability to predict death and AKI-D was validated by discriminability and fit in the general population, patients with elective cardiac surgery, or non-elective cardiac surgery. Discriminability was determined by the area under the receiver operating characteristics (ROC) curve (AUC) (> 0.7). The comparison of AUCs between the scores was determined by the rank-sum test described by DeLong et al. [31]. A calibration curve was drawn based on the predicted and actual values and evaluated by the Hosmer–Lemeshow test. *P*-values < 0.05 were considered statistically significant.

## Results

### Characteristics of the patients

**Fig. 1:**
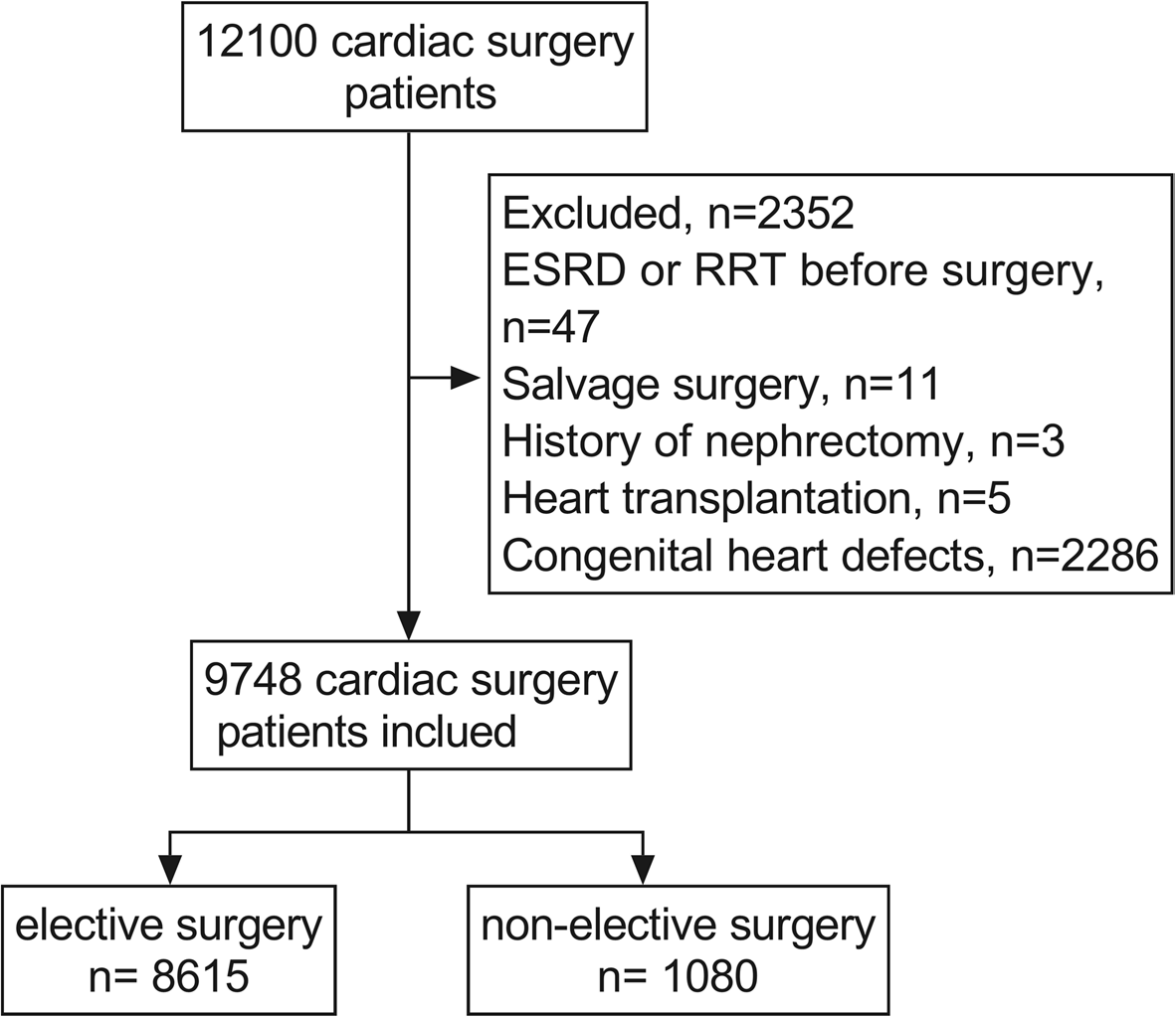
Flowchart of study population selection

This study examined 12,100 patients for eligibility; 2352 were excluded according to the exclusion criteria, and 9748 patients were included (Fig. 1). Among them, 1080 patients underwent non-elective surgery within 24 h after admission, and the remaining 8615 patients underwent elective surgery. The in-hospital mortality was 1.8% (177/9748). The incidence of AKI-D was 3.5% (345/9748). The in-hospital mortality was 1.2% (105/8615) in the elective patients and 6.6% (72/1080) in the non-elective patients. The incidence of AKI-D was 2.6% (227/8615) and 10.9% (118 /1080) in elective and non-elective patients, respectively. The ACEF and ACEF II scores of the patients in the elective surgery group were 0.86 ± 0.30 and 1.01 ± 0.52 and were 0.89 ± 0.37 and 2.08 ± 1.60 in the non-elective surgery group. The patients’ demographic and clinical characteristics are shown in Table 1. The patients with non-elective surgery had a higher proportion of hypertension, hematocrit < 36%, and preoperative critical condition (*P* < 0.05).

**Table 1:** Characteristics of the patients

Variable	Total (*n* = 9748)	Elective surgery (*n* = 8615)	Non-elective surgery (*n* = 1080)	*P*
Age (years)	51.4 ± 12.5	51.4 ± 12.4	51.6 ± 12.9	0.631
Female	4566 (46.8)	4176 (48.2)	390 (35.9)	< 0.001
Hypertension	2185 (22.4)	1780 (20.6)	405 (37.3)	< 0.001
Diabetes mellitus on insulin	556 (5.7)	483 (5.6)	73 (6.7)	0.127
CKD	53 (0.5)	46 (0.5)	7 (0.6)	0.633
COPD	258 (2.6)	216 (2.5)	42 (3.9)	0.008
CCS class IV angina^a^	213 (2.2)	181 (2.1)	32 (2.9)	0.069
Recent myocardial infarction^a^	95 (1.0)	63 (0.7)	32 (2.9)	< 0.001
Extracardiac arteriopathy^a^	74 (0.8)	60 (0.7)	14 (1.3)	0.033
Poor mobility^a^	88 (0.9)	73 (0.8)	15 (1.4)	0.078
Previous cardiac surgery	360 (3.7)	315 (3.6)	45 (4.1)	0.407
Ejection fraction (%)	61.96 ± 9.37	61.98 ± 9.30	61.77 ± 9.92	0.504
Ejection fraction (%)				0.202
Normal (> 50%)	8740 (89.7)	7767 (89.7)	973 (89.5)	
Mild/moderate (30%-50%)	968 (9.9)	862 (10.0)	106 (9.8)	
Severe (< 30%)	40 (0.4)	32 (0.4)	8 (0.7)	
Serum creatinine (mg/dL)	0.9 ± 0.3	0.94 ± 0.28	1.02 ± 0.35	< 0.001
Serum creatinine > 2.0 mg/dL	41 (0.4)	26 (0.3)	15 (1.4)	< 0.001
Serum creatinine ≥ 2.0 mg/dL	69 (0.7)	48 (0.6)	21 (1.9)	< 0.001
eGFR	85.5 ± 22.6	85.94 ± 22.30	81.93 ± 24.55	< 0.001
Hematocrit < 36%	2222 (22.8)	1824 (21.1)	398 (36.6)	< 0.001
Critical preoperative state^a^	276 (2.8)	202 (2.3)	74 (6.8)	< 0.001
Procedure				< 0.001
CABG alone	887 (9.1)	730 (8.4)	157 (14.4)	
Valve surgery alone	6039 (62.0)	5592 (64.6)	447 (41.4)	
Thoracic aorta alone	425 (4.4)	233 (2.7)	192 (17.7)	
Complex procedures	2397 (24.6)	2106 (24.3)	291 (26.8)	
CPB time	123.18 ± 65.42	120.81 ± 61.67	141.99 ± 87.55	< 0.001
postoperative LCOS^a^	119 (1.2)	94 (1.1)	25 (2.3)	0.001
Postinfarct septal rupture^a^	47 (0.5)	36 (0.4)	11 (1.0)	0.007
In-hospital mortality	177 (1.8)	105 (1.2)	72 (6.6)	< 0.001
Renal replacement therapy	345 (3.5)	227 (2.6)	118 (10.9)	< 0.001
ACEF score	0.87 ± 0.31	0.86 ± 0.30	0.89 ± 0.37	0.004
ACEF II score	1.1 ± 0.8	1.01 ± 0.52	2.08 ± 1.60	< 0.001

*CKD* Chronic kidney disease, *COPD* Chronic obstructive pulmonary disease, *CCS* Canadian Cardiovascular Society, eGFR estimated glomerular filtration rate, *PA* Pulmonary artery, *IABP* Intra-aortic balloon pump, *CABG* Coronary artery bypass grafting, *CPB* Cardiopulmonary bypass, *LCOS* low cardiac output syndrome, *ACEF* Age, creatinine and ejection fraction

^a^The variable was defined according to the Euro SCORE II definitions

### Prediction of the risk of in-hospital death

**Fig. 2:**
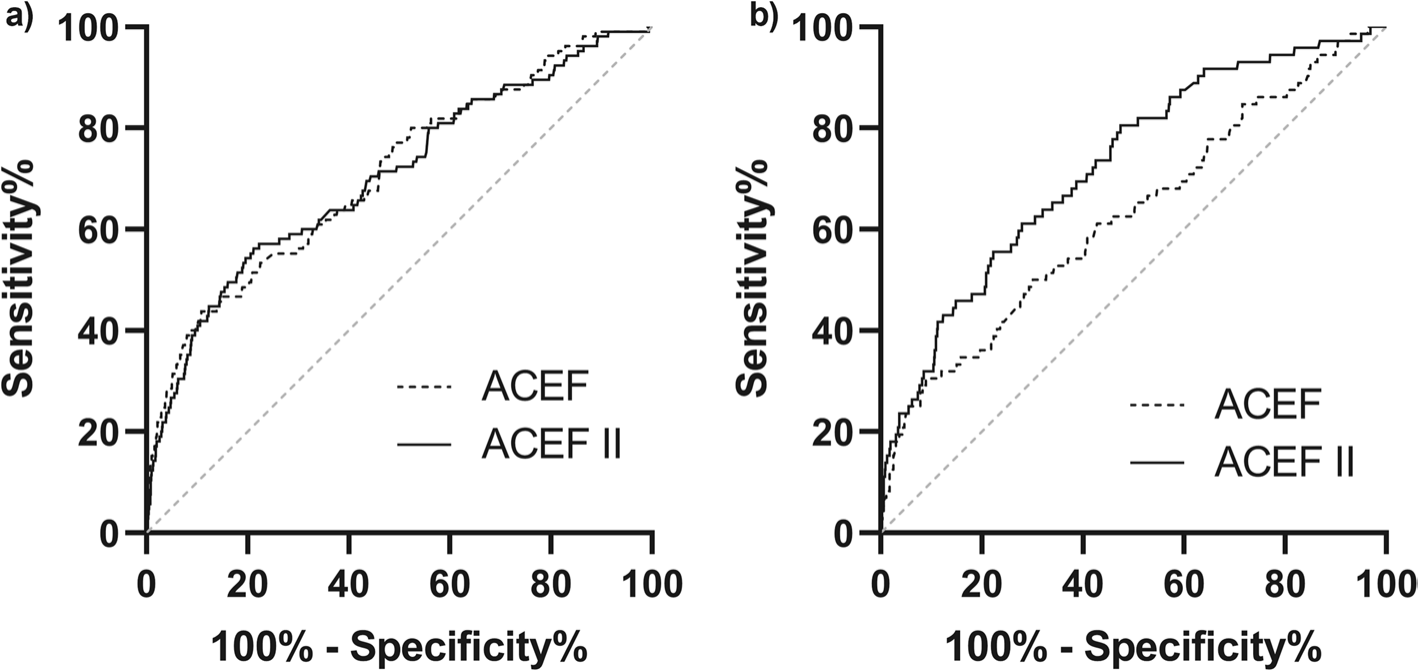
Comparison of the area under the receiver operating characteristic curves (AUROCs) among models for mortality. **a** Elective cardiac surgery. ACEF score AUROC: 0.709 (95%CI 0.654–0.763) vs. ACEF II score: 0.704 (95%CI 0.648–0.759), *P* = 0.788. **b** Non-elective cardiac surgery. ACEF score AUROC: 0.625 (95%CI 0.553–0.697) vs. ACEF II score: 0.725 (95%CI 0.663–0.787), *P* = 0.020

**Fig. 3:**
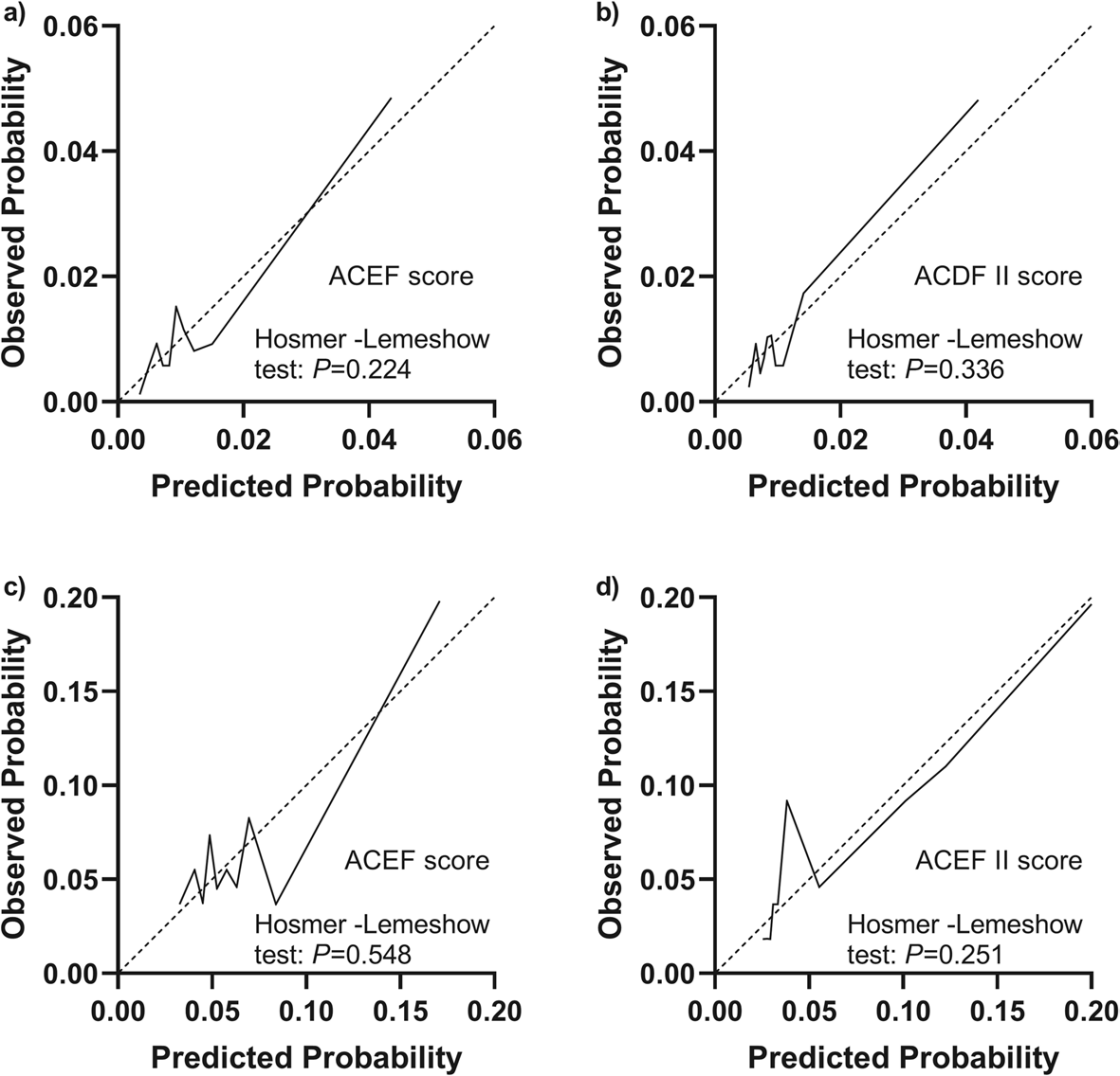
Calibration curves of the ACEF and ACEF II scores for predicting the risk of mortality. **a**, **b** Elective cardiac surgery. **c**, **d** Non-elective cardiac surgery

The AUCs of the ACEF and ACEF II scores for predicting death for all cardiac surgery patients were 0.675 (95%CI: 0.631–0.719) and 0.755 (95%CI: 0.714–0.796) (Fig. 2). The ACEF and ACEF II scores were 0.709 (95%CI 0.654–0.763) and 0.704 (95%CI 0.648–0.759) in elective cases, and 0.625 (95%CI 0.553–0.697) and 0.725 (95%CI 0.663–0.787) in non-elective cases. The calibration curves indicated that ACEF and ACEF II had goodness of fit in predicting death in elective surgery, and ACEF II had goodness of fit in predicting death in non-elective cases (Fig. 3), as evaluated by the Hosmer–Lemeshow test (*P* > 0.05).

### Anticipating the risk of AKI-D

**Fig. 4:**
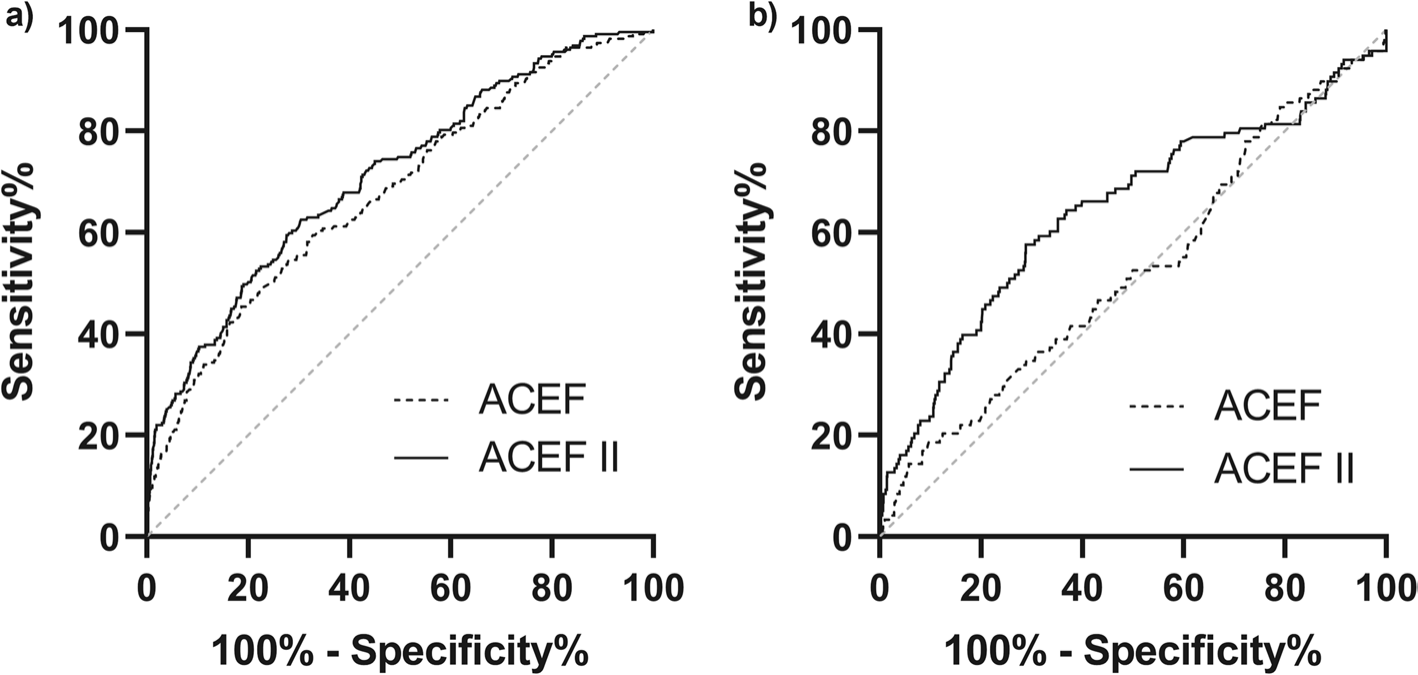
Comparison of the area under the receiver operating characteristic curves (AUCs) among models for acute kidney injury requiring renal replacement therapy. **a** Elective cardiac surgery. ACEF score AUC: 0.678 (95%CI 0.641–0.715) vs. ACEF II score AUC: 0.711 (95%CI 0.675–0.747), *P* = 0.043. **b** Non-elective cardiac surgery. ACEF score AUC: 0.524 (95%CI 0.467–0.582) vs. ACEF II score AUC: 0.642 (95%CI 0.583–0.702), *P* < 0.001

**Fig. 5:**
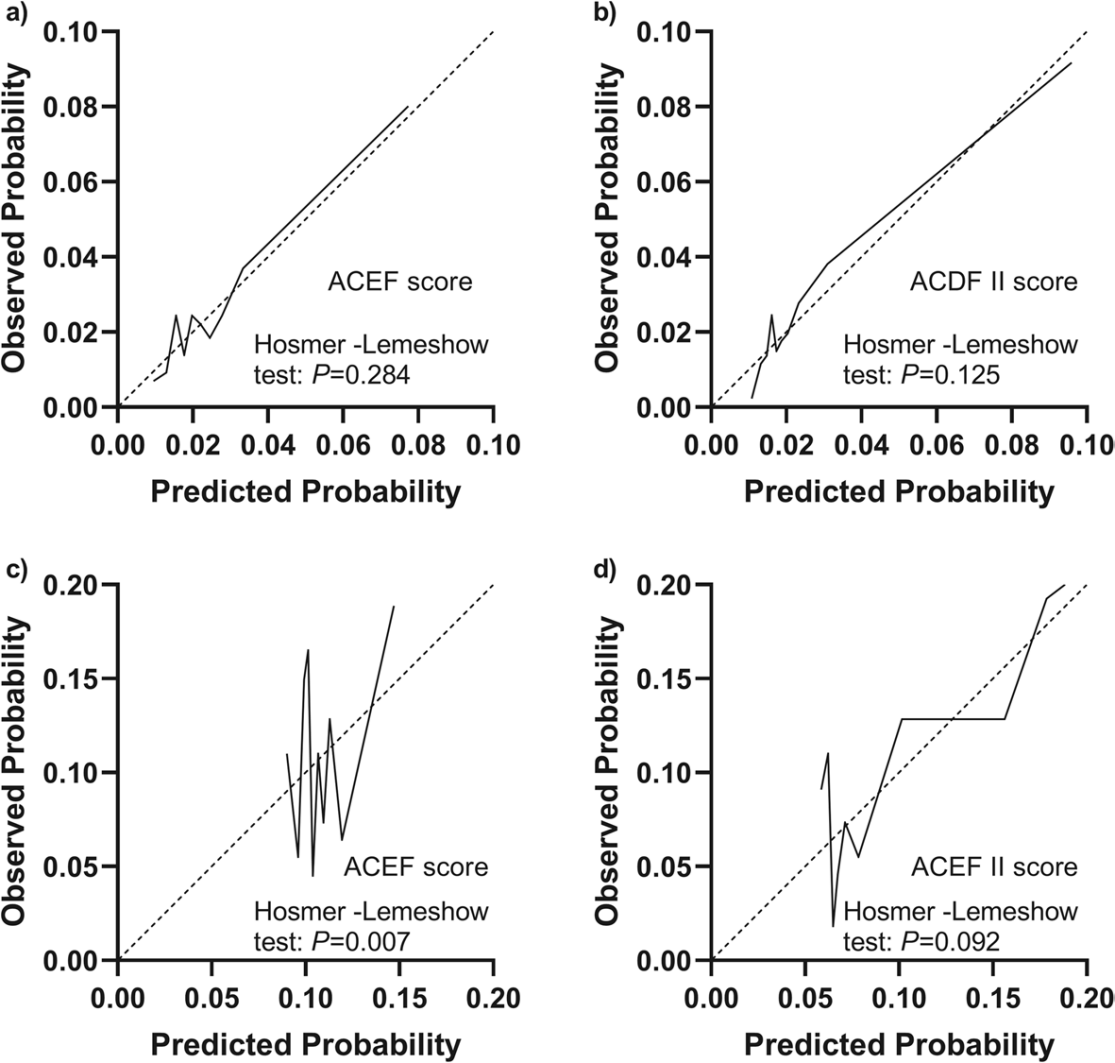
Calibration curves of ACEF score and ACEF II score for predicting the risk of RRT, respectively. **a**, **b** Elective cardiac surgery. **c**, **d** Non-elective cardiac surgery

The AUCs predicting AKI-D were 0.626 (95% CI 0.594–0.658) and 0.718 (95% CI 0.687–0.749) (Fig. 4). The scores were 0.678 (95% CI 0.641–0.715) and 0.711 (95% CI 0.675–0.747) in elective cases, and 0.524 (95% CI 0.467–0.582) and 0.642 (95% CI 0.583–0.702) in non-elective cases. The calibration curves showed that the ACEF II score had goodness of fit in AKI-D after elective surgery, as evaluated by The Hosmer–Lemeshow test (*P* > 0.05) (Fig. 5).

## Discussion

Risk assessment before cardiac surgery might improve prognosis. Mortality after cardiac surgery has declined in recent decades [10], but AKI-D remains a risk factor for postoperative mortality. Still, the morbidity of AKI-D has not decreased significantly [32, 33] due to the increasing proportion of elderly patients with more comorbidities and complications [34]. The risk factors included in the ACEF and ACEF II models for predicting post-surgery mortality are also risk factors of AKI, and the ACEF can be used to predict postoperative AKI.

Chen SW et al. [23]explained that ACEF can be used to predict all stages of AKI. Chang CH et al. [24] demonstrated that ACEF scores exhibited satisfactory predictive ability for all AKI severities.

Nevertheless, the existing AKI-D models after cardiac surgery have a poor discriminative ability in Chinese with different disease spectrums, mainly because there were established based on European and North American populations. In addition, ACEF is mainly aimed at the elective surgery population and is not suitable for emergency patients. The ACEF II model was developed to adapt the ACEF to emergency surgery, but the performance of ACEF II in predicting death and AKI-D in the Chinese population is unclear. Hence, this study aimed to evaluate the value of the ACEF II score in predicting postoperative hospital death and AKI-D. The results indicate that ACEF and ACEF II have poor discriminative ability in predicting AKI-D in non-elective surgery. ACEF II and ACEF scores have the same ability to predict in-hospital death in elective cardiac surgery, and the ACEF II score is better in non-elective surgery. The ACEF II score can be used to assess the risk of AKI-D in elective surgery.

Several models can predict AKI-D after cardiac surgery [15–17]. The Cleveland Risk Score [17] includes 10 variables and is based on the data of 15,838 patients who underwent cardiac surgery, including 68.9% with coronary bypass surgery without valve surgery, which is a procedure increasingly used in China, ranging from 55.1% to 70% [35, 36]. Still, external verification showed poor performance for valve surgery patients [35, 37], and there is no model to evaluate AKI-D for Chinese patients. The EuroSCORE II [18], STS score [19], and ACEF score [20] are commonly used in clinical practice to identify patients at risk of AKI and/or death after open-heart surgery. Still, they have disadvantages. Indeed, the EuroSCORE II [18] includes 17 variables and was established based on 22,381 patients from multiple centers. The STS score [19] includes 42 risk factors based on various heart surgery strategies. Hence, the EuroSCORE II and STS scores require assessing large numbers of factors and variables and complicated calculations, making them arduous to use in the routine clinical setting. The much simpler ACEF score [20] includes only three variables (age, ejection fraction, and serum creatinine) [21] and can quickly and easily assess the risk of 30-day death and AKI-D in elective cardiac surgery [15–17], but not in non-elective surgery [9]. The ACEF score could predict severe AKI after coronary artery bypass grafting [23]. Therefore, Ranucci et al. [22] established the ACEF II score by adding emergency surgery and anemia to the ACEF score.

Still, the ACEF and ACEF II scores were not assessed in Chinese patients undergoing elective or non-elective surgery. In the present study, the ACEF II and ACEF scores were calculated to predict in-hospital death in the elective cardiac surgery group. The ACEF II score was better than the ACEF score in the non-elective surgery group. In addition, the ACEF II score can be used to predict the risk of AKI-D in patients with elective cardiac surgery. Still, it should be used with caution in patients with non-elective cardiac surgery. The results showed that the ACEF score could predict the risk of death in patients undergoing elective cardiac surgery. Still, the discrimination of the ACEF score is low in the non-elective surgery group (the area under the ROC curve was < 0.7), which is similar to the results of previous studies [9, 21]. It is probably because the ACEF score was originally established based on the clinical data of a non-elective cardiac surgery population. Indeed, patients with non-elective surgery have a higher proportion of preoperative critical conditions and complicated surgery because of various comorbidities. Non-elective surgery is also an independent risk factor for death after cardiac surgery. The ACEF II and ACEF scores have three common variables with the same weights. The results show that the ability of the ACEF II score to assess death was similar to the ACEF score in the elective surgery population. The ACEF II score includes two supplementary variables (emergency surgery and hematocrit < 36%), which are independent risk factors for death after cardiac surgery [18, 38], as also observed in the present study (Supplementary Table S1). The ACEF II score has also been developed to predict death in patients with non-elective surgery. Although non-elective surgery accounts for a low proportion, their condition is complicated and fatal if not treated in time.

The incidence of AKI-D was 3.5%, higher than the average incidence in the previous studies [37, 39], which might be related to the following reasons. The distribution of medical resources in China is still uneven, and the early diagnosis rate is lower than in Europe and North America. In addition, basic heart surgery is mainly for valvular disease in China [35]. Studies have confirmed that complicated cardiac surgery and cardiopulmonary bypass (CPB) time are independent risk factors for AKI-D [15, 40]. The proportion of complicated heart surgery in Chinese is higher than that of coronary artery disease in Europe and North America (24.6 vs. 19%), and the CPB time was longer than in Europe and North America (123 ± 65.42 vs. 100 ± 36 min) [15]. Finally, the timing to start dialysis treatment is affected by the subjective factors of the consulting physicians, which also affects the incidence.

The variables of the ACEF II score are independent risk factors for AKI-D after cardiac surgery [15–17, 41]. Therefore, the results showed that the ability of the ACEF II score to assess AKI-D after non-elective cardiac surgery is acceptable, but not after elective cardiac surgery. The variables of the scoring model are simple, and the condition of non-elective surgery is more complicated. The occurrence of AKI-D might be related to the preoperative critical status, neurological dysfunction, and hypertension, which are independent risk factors for AKI-D after cardiac surgery [39]. It is necessary to establish a scoring system predicting AKI-D suitable for the non-elective cardiac surgery Chinese population.

There are some limitations to this study. First, the retrospective data from a single center inevitably result in some bias, limiting the generalizability of the results. In addition, the mortality was in-hospital death, which might be underestimated due to Chinese customs. Finally, because the calculation of the EuroSCORE II and STS scores requires many variables and complex calculations, those scores could not be calculated because of missing data in most patients. Therefore, only the ACEF could be used as a comparator.

## Conclusion

The ACEF II score can be used to distinguish between the low- and high-risk groups of postoperative deaths in patients undergoing elective and non-elective cardiac surgery and provides a clinical score to predict AKI-D in elective cardiac surgery. ACEF and ACEF II have poor discrimination ability in predicting AKI-D in non-elective surgery. ACEF II and ACEF scores have the same ability to predict in-hospital death in elective cardiac surgery, and the ACEF II score is better in non-elective surgery. In addition, the variables of the ACEF II score are easy to obtain, and the score is simple to calculate. Therefore, it can be used in the clinic to optimize the choice of treatment plans and facilitate the allocation of medical resources to Chinese patients.

## Supplementary Information

**Additional file 1: Supplementary Table 1.** Logistic regression analysis of preoperative variables for mortality in all cardiac surgery.

## Data Availability

The datasets used and/or analysed during the current study are available from the corresponding author on reasonable request.
