# Supplementary material for: The value of the ACEF II score in Chinese patients with elective and non-elective cardiac surgery
Source: BMC Cardiovasc Disord. 2022 Dec 2;22:513. doi: 10.1186/s12872-022-02946-6 (PMC9716978; doi:10.1186/s12872-022-02946-6)
Supplement: Supplementary file 1 — Additional file 1: Supplementary Table 1. Logistic regression analysis of preoperative variables for mortality in all cardiac surgery. [file 12872_2022_2946_MOESM1_ESM.docx]

**Supplementary Table 1:** Logistic regression analysis of preoperative variables for mortality in all cardiac surgery

| Variable | β-Coefficient | SE | OR | 95%CI | p-value |
| --- | --- | --- | --- | --- | --- |
| Univariate logistic regression |  |  |  |  |  |
| Age (years) | 0.039 | 0.007 | 1.040 | 1.026 - 1.054 | <0.001 |
| Female | -0.500 | 0.159 | 0.607 | 0.444 - 0.828 | 0.002 |
| Hypertension | 0.762 | 0.157 | 2.143 | 1.576 - 2.915 | <0.001 |
| Diabetes mellitus on insulin | 0.276 | 0.292 | 1.318 | 0.744 - 2.334 | 0.344 |
| CKD | 1.189 | 0.599 | 3.283 | 1.014 - 10.627 | 0.047 |
| COPD | 0.260 | 0.420 | 1.298 | 0.569 - 2.957 | 0.535 |
| CCS class IV angina^a^ | 1.016 | 0.333 | 2.763 | 1.438 - 5.309 | 0.002 |
| Recent myocardial infarction^a^ | 0.152 | 0.719 | 1.165 | 0.285 - 4.764 | 0.832 |
| Extracardiac arteriopathy^a^ | 0.836 | 0.594 | 2.307 | 0.720 - 7.395 | 0.160 |
| Poor mobility^a^ | 1.862 | 0.360 | 6.437 | 3.177 - 13.043 | <0.001 |
| Previous cardiac surgery | 1.180 | 0.249 | 3.255 | 1.998 - 5.303 | <0.001 |
| Ejection fraction (%) | -0.047 | 0.007 | 0.955 | 0.942 - 0.967 | <0.001 |
| Serum creatinine >2.0mg/dL | 3.048 | 0.361 | 21.074 | 10.385 - 42.766 | <0.001 |
| Haematocrit <36% | 0.980 | 0.154 | 2.666 | 1.973 - 3.602 | <0.001 |
| Critical preoperative state^a^ | 2.624 | 0.185 | 13.784 | 9.591 - 19.809 | <0.001 |
| Procedure type | 0.281 | 0.074 | 1.324 | 1.145 - 1.532 | <0.001 |
| Postinfarct septal rupture^a^ | 0.883 | 0.727 | 2.419 | 0.582 - 10.051 | 0.224 |
| Urgency^a^ |  |  |  |  |  |
| Urgent | 1.320 | 0.201 | 3.744 | 2.525 - 5.552 | <0.001 |
| Emergency | 2.421 | 0.199 | 11.260 | 7.624 - 16.630 | <0.001 |
| Multivariate logistic regression |  |  |  |  |  |
| Age (years) | 0.045 | 0.008 | 1.046 | 1.031 - 1.062 | <0.001 |
| Poor mobility^a^ | 1.665 | 0.408 | 5.287 | 2.378 11.757 | <0.001 |
| Previous cardiac surgery | 1.445 | 0.27 | 4.242 | 2.499 - 7.200 | <0.001 |
| Ejection fraction (%) | -0.044 | 0.007 | 0.957 | 0.944 - 0.971 | <0.001 |
| Serum creatinine >2.0mg/dL | 2.129 | 0.459 | 8.408 | 3.421 - 20.669 | <0.001 |
| Haematocrit <36% | 0.631 | 0.172 | 1.879 | 1.341 - 2.632 | <0.001 |
| Critical preoperative state^a^ | 2.194 | 0.207 | 8.972 | 5.982 - 13.456 | <0.001 |
| Procedure type | 0.201 | 0.078 | 1.222 | 1.049 - 1.424 | 0.010 |
| Urgency |  |  |  |  |  |
| Urgent | 0.969 | 0.217 | 2.635 | 1.723 - 4.028 | <0.001 |
| Emergency | 2.224 | 0.234 | 9.247 | 5.842 - 14.637 | <0.001 |

Abbreviations: CKD,chronic kidney disease; COPD, chronic obstructive pulmonary disease; CCS, Canadian Cardiovascular Society.

^a^ The variable was defined according to the EuroSCORE II definitions.
